# Disparate nonlinear neural dynamics measured with different techniques in macaque and human V1

**DOI:** 10.1038/s41598-024-63685-6

**Published:** 2024-06-08

**Authors:** Jingyang Zhou, Matt Whitmire, Yuzhi Chen, Eyal Seidemann

**Affiliations:** 1 Center for Computational Neuroscience, Flatiron Institute, New York, USA; 2 Center for Neural Science, New York University, New York, USA; 3 Center for Perceptual Systems, University of Texas, Austin, Austin, USA; 4 Center for Theoretical and Computational Neuroscience, University of Texas, Austin, Austin, USA; 5 Department of Psychology, University of Texas, Austin, Austin, USA; 6 Department of Neuroscience, University of Texas, Austin, Austin, USA

**Keywords:** Neuroscience, Physiology

## Abstract

Diverse neuro-imaging techniques measure different aspects of neural responses with distinct spatial and temporal resolutions. Relating measured neural responses across different methods has been challenging. Here, we take a step towards overcoming this challenge, by comparing the nonlinearity of neural dynamics measured across methods. We used widefield voltage-sensitive dye imaging (VSDI) to measure neural population responses in macaque V1 to visual stimuli with a wide range of temporal waveforms. We found that stimulus-evoked VSDI responses are surprisingly near-additive in time. These results are qualitatively different from the strong sub-additive dynamics previously measured using fMRI and electrocorticography (ECoG) in human visual cortex with a similar set of stimuli. To test whether this discrepancy is specific to VSDI—a signal dominated by subthreshold neural activity, we repeated our measurements using widefield imaging of a genetically encoded calcium indicator (GcaMP6f)—a signal dominated by spiking activity, and found that GCaMP signals in macaque V1 are also near-additive. Therefore, the discrepancies in the extent of sub-additivity between the macaque and the human measurements are unlikely due to differences between sub- and supra-threshold neural responses. Finally, we use a simple yet flexible delayed normalization model to capture these different dynamics across measurements (with different model parameters). The model can potentially generalize to a broader set of stimuli, which aligns with previous suggestion that dynamic gain-control is a canonical computation contributing to neural processing in the brain.

## Introduction

Neural nonlinearities play an important role in shaping our rich and flexible visual perception. Neural representations throughout the primate visual system display a wide range of nonlinear dynamics to visual stimuli^1–8^. To understand the neural basis of visual perception, it is important to quantify the neural response dynamics and to characterize the nonlinearities within these dynamics.

The representations of visual stimuli in primate V1 (primary visual cortex) are widely distributed: a small and localized stimulus activates millions of neurons distributed over more than 10 mm^2^ in a macaque’s V1^9,10^. No current technique can measure activities of all neurons that may be perceptually relevant with single-cell resolution and real-time dynamics in V1 of a behaving primate. Therefore, to understand the neural basis of visual perception, we need to combine multiple complementary techniques, each of which samples a different aspect of brain activity at a different spatial and temporal resolution. Here, we take a step towards this long-term goal, by comparing how neurons dynamically respond to visual stimuli in primate visual cortex across different measurement methods and across different spatial and temporal scales.

Neural dynamics measured using different methods can exhibit different properties. This is because different measurement methods emphasize different signals within a neural population. For example, widefield voltage-sensitive dye imaging (VSDI) signals are dominated by spatially pooled membrane potentials of cortical neurons^9,10^, while widefield GCaMP signals seem to link more closely to pooled spiking activities^11^. fMRI is the most prevalent method used to measure behaving humans’ brain responses. fMRI indirectly measures neural activities, and it has been challenging to relate the fMRI BOLD signals to a local neural population’s stimulus-evoked responses^12–15^.

In this study, we compared neural dynamics measured using different methods. We first used VSDI to measure population responses from V1 of behaving macaques to large and high-contrast visual stimuli that were presented using a range of time courses. Our first set of goals was to assess the degree of nonlinearity in these responses, and to develop a computational model that can predict the dynamics of V1 responses to an image presented with arbitrary temporal waveforms. Our second goal was to compare temporal additivity measured using VSDI to those measured using fMRI and ECoG in human visual cortex^15,16^. We found that stimulus-evoked dynamics measured using these methods have qualitatively different properties. Stimulus-evoked VSDI dynamics measured in behaving Macaques’ V1 were near-additive over a wide range of stimulus time courses, while fMRI and ECoG dynamics measured for similar stimuli in human V1 were strongly sub-additive in time.

To test whether this difference is due to disparate spiking versus subthreshold neural population dynamics, we additionally measured widefield GCaMP responses to the same set of temporal conditions in behaving macaque V1. We found that like VSDI, GCaMP signals were also near-additive, suggesting that the difference in sub- vs. supra-threshold dynamics are unlikely the reason why nonlinearities measured in fMRI, ECoG and in VSDI are different.

Finally, we show that a simple delayed normalization model can qualitatively account for the dynamics of all these measurements, suggesting that dynamic gain-control is likely to be an important mechanism contributing to neural processing in the brain.

## Results

### Temporal additivity in stimulus-evoked VSDI dynamics

**Figure 1:**
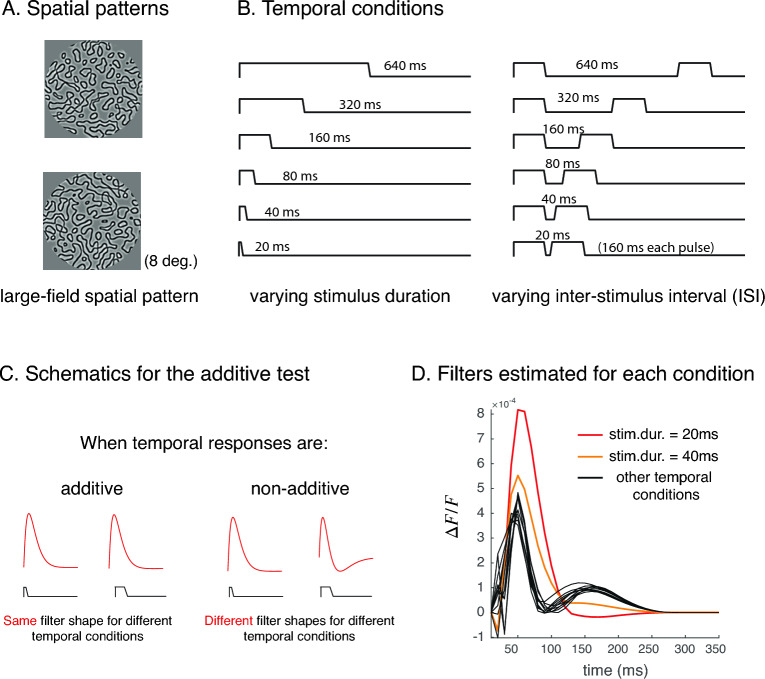
Stimulus and additivity test. (**A**) Subjects (monkeys) viewed large patterned stimuli that strongly activated a population of V1 neurons. (**B**) Subjects were presented with a single pulse of an image with 6 different durations, and double pulses of an image (i.e. repeated presentations) with 6 different inter-stimulus-intervals (ISIs). (**C**) For each of the 12 temporal conditions, we estimated a different filter from the trial-averaged response time course. If neural responses were linear (or were additive in our case), impulse response functions estimated for each temporal condition would share the same shape. If filters estimated for different conditions had different shapes, the stimulus-evoked responses cannot be linear. (**D**) Other than when stimulus durations were brief, filters estimated from stimulus-evoked VSDI responses were very similar in shape.

To assess properties of stimulus-evoked VSDI responses, we presented a set of large static patterned images (band-pass filtered noise peaking at 3 cpd, 100% contrast^15^) with different temporal conditions (Fig. 1A) while monkeys performed a fixation task. The first type of temporal conditions was a single pulse of image presented using 6 different durations. The presentation durations ranged from 20 to 640 ms, and the duration in each condition was twice as long as the previous condition (20, 40, 80, 160, 320, 640 ms). The second type of temporal conditions was a single image presented twice, each for 160 ms with different inter-stimulus intervals (ISIs). The ISIs range from 20 to 640 ms, and like varying durations, each ISI was twice as long as the previous ISI (Fig. 1B). Because the stimulus was larger than the portion of the visual field represented in our imaging cranial windows, it elicited a response that was spatially uniform within the imaged area. We focus on analyzing the time course of the response within an area of several square mm within the center of the imaging chamber.

To analyze stimulus-evoked VSDI dynamics, we first assessed whether these dynamical responses to the two sets of temporal conditions can be explained by a linear model. Raw VSDI dynamics consist of two components^17,18^. The first component is relatively fast, and closely tracks the stimulus time course $s\left(t\right)$. This component is related to stimulus-evoked population membrane potential responses^19,20^. We modeled this component using a linear filter $f\left(t\right)$ convolved with the stimulus time course $s\left(t\right)$. The second component of the VSDI time courses is a slow-varying compound signal that is likely to reflect a mixture of neural and non-neural slow variability. We modeled this component using a slow-varying function $g\left(t\right)$, which is at least an order of magnitude slower than the first component^21,22^. Overall, the linear prediction to the VSDI dynamics ${r}_{l}(t)$ can be summarized by additively combining the two components:

$${r}_{l}(t)= s\left(t\right)*f\left(t\right)+g(t)$$

In the Supplement, we show that $g\left(t\right)$ captured slow variations in the signal that were largely stimulus independent (Figs. S1, S2). To understand how the fast and stimulus-evoked component $f\left(t\right)$ varies across stimulus conditions, we removed $g\left(t\right)$ from the raw VSDI time courses, and used the remaining data for further analyses. For more details of data processing, see the “Methods” section.

To quantify stimulus-evoked VSDI dynamics (the fast component), we first assessed how and to what extent the extracted stimulus-evoked responses deviate from the predictions of a linear model. To do so, for each temporal condition, we fitted a linear model (with potentially a different set of parameters) to the trial-averaged data, and we estimated a separate temporal filter for each condition. If the stimulus-evoked dynamics were linear, the filters fit to different temporal conditions would share the same shape, because a single filter uniquely characterizes a linear system (Fig. 1C). If the estimated filters had different shapes, the system would be nonlinear and through observing the differences between filters, we can study the types of nonlinearities that exist in the data (e.g.^23^).

The VSDI filter shapes estimated for different stimulus conditions were similar to those estimated in single-cell membrane potentials^24–26^. Surprisingly, the estimated filters for all temporal conditions beyond the two shortest durations shared a similar shape, suggesting that the pooled membrane potential in V1 is nearly additive in time for stimulus duration equal to or longer than 80 ms. For brief stimulus durations (< 80 ms), estimated filters tend to be larger in gain (higher in amplitude), monophasic (rather than biphasic), and have slower dynamics (Fig. 1D). For example, the estimated time to peak for filters was around 50 ms for brief stimulus durations (excluding the latency between stimulus onsets and response onsets), and was around 40 ms for longer-duration stimuli. Our observation confirmed that stimulus-evoked VSDI responses deviate from the linear prediction, and the deviation is possibly due to gain adjustment that depends on stimulus duration. This observation is consistent with duration-dependent gain changes observed in neural responses measured using other methods and in other parts of visual pathways^16,27,28^. In a later section, we compare how much temporal dynamics deviate from the additive predictions across measurement methods.

### Capturing non-linear VSDI dynamics using a delayed normalization model

**Figure 2:**
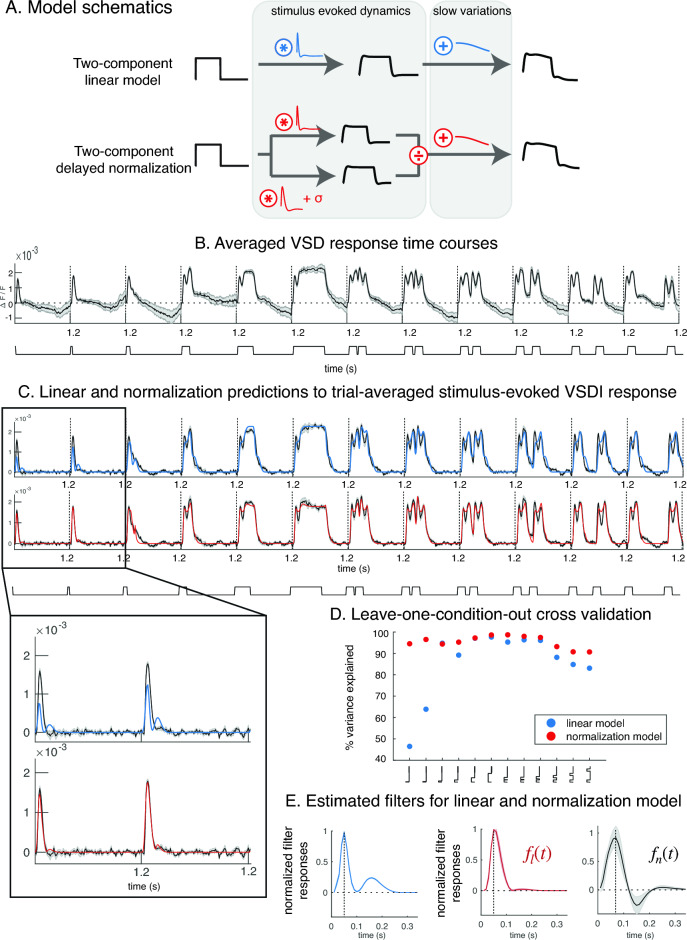
Fitting linear and delayed normalization models to VSDI dynamics. (**A**) The linear model consists of a fast component (a filter convolved with a stimulus time course) that captures stimulus-evoked dynamics. The delayed normalization also consists of a divisive computation that intends to capture the same dynamics in VSDI measurements. (**B**) The averaged VSDI data dynamics (from one animal) in response to the twelve temporal conditions. The black curve indicates the VSDI data averaged across eight stimulus repeats (seven for 2 stimulus conditions, see Fig. S1). The shaded area indicates standard error, computed by bootstrapping the averaged dynamics (sampling with replacement over 50 bootstraps). The stimulus time course was plotted below the data panel. (**C**) Delayed normalization model, compared to the linear model, better captured stimulus-evoked VSDI dynamics when stimulus presentations were brief. Both models were fit to each bootstrap of the mean response dynamics. Both model-predicted fast components of the VSDI data were plotted, the linear model in blue, and delayed normalization in red. (**D**) Leave-one-condition-out cross validation further confirms that the delayed normalization improved upon the linear model at brief and long stimulus durations. The cross-validation was performed on the trial-averaged data.  (**E**). Estimated filter for linear, and for normalization model (${f}_{l}(t)$). Both the filter for the linear model (blue), and ${f}_{l}(t)$ for the normalization model (in red) are similar in terms of their time to peak, but ${f}_{l}(t)$ tends to be simpler in shape. This is because some of the dynamics captured by the linear filter was absorbed into the normalization dynamics. The filter time to peak for the normalization dynamics (denominator of the delayed normalization model, in black) is delayed compared to ${f}_{l}(t)$, and ${f}_{n}(t)$ is biphasic in shape (black).

We used a delayed normalization model to account for gain changes in the stimulus-evoked VSDI responses to brief stimuli^4^. The model has a divisive form, and it consists of a numerator and a denominator, each involves a linear computation. The model numerator consists of a filter ${f}_{l}(t)$ convolved with a stimulus time course, $s\left(t\right)*{f}_{l}\left(t\right)$. The model denominator consists of another filter ${f}_{n}(t)$ convolved with the same stimulus time course, $s\left(t\right)*{f}_{n}(t)$. A positive constant σ is added to the denominator to prevent computations from becoming undefined when stimulus time course is 0, which is the case when stimulus contrast is zero (Fig. 2A). The delayed normalization model intends to summarize the stimulus-evoked component in the VSDI signal. Moreover, we additively combined the delayed normalization with $g(t)$ to account for slow variations in the measurement time series. We fit the combined model to the overall VSDI dynamics to reduce estimation bias, and the combined model prediction ${r}_{n}(t)$ has the following form:

$${r}_{n}(t) = \frac{s\left(t\right)*{f}_{l}(t)}{\sigma +s\left(t\right)*{f}_{n}(t)}+g\left(t\right)$$

A single set of delayed normalization parameters ${\{f}_{l}\left(t\right), {f}_{n}\left(t\right), \sigma \}$ was fit to all temporal conditions in the trial-averaged data and captured the fast stimulus evoked response across all 12 stimulus conditions. The slow residual variability was captured by $g(t)$, which was fit separately for each temporal condition, so 12 sets of slow components were fit to the 12 temporal conditions (see “Methods” and Fig. S2).

To understand whether and to what extent the delayed normalization model improved upon a linear model, we compared the delayed normalization model to the linear model with a single filter fit across all temporal conditions (Fig. 2A). The delayed normalization model improved upon the linear model, especially when stimulus durations were brief (Fig. 2C). We can infer why the delayed normalization model improved the fit by examining how the model works. At stimulus onsets, the model numerator starts to respond, and this linear computation dominates the response dynamics before normalization dynamics kick in. Because the denominator filter is delayed compared to the numerator filter, after a brief time period, the normalization dynamics start to strengthen, and decrease the response gain while speeding up the dynamics. The temporal difference between the stimulus-drive (numerator) and the normalization drive (denominator) can account for our stimulus-evoked VSDI responses—dynamics are slower and the gain is higher for brief stimulus presentations.

The delayed normalization model performed better than the linear model in leave-one-condition-out cross-validations (Fig. 2D). This type of cross-validation examines whether fitted model parameters well-generalize to predict response dynamics in a different stimulus condition. We further investigated the filter shape estimated using delayed normalization (Fig. 2E). The filter estimated for the stimulus-drive (numerator filter) was comparable to the linear model filter in terms of the time to peak (50 ms for both the linear model filter and the delayed normalization numerator filter). Compared to the linear model filter, the numerator filter of the normalization model tended to have a simpler (and more monophasic) shape. This is because some dynamics of the linear model filter were absorbed into the normalization dynamics. In the delayed normalization model, the peak of the denominator filter is around 70 ms, which is delayed compared to the numerator filter time-to-peak, and the estimated denominator filter has a biphasic shape (and the filter’s second negative peak around 150 ms) (Fig. 2E). The second peak predicted by the linear model could be explained by the shape of its filter (stimulus drive), whereas in delayed normalization, the second peak was explained by the suppressive effect from its denominator filter.

### Disparate temporal additivities measured using different methods

In the previous section, we accounted for stimulus-evoked VSDI dynamics using a delayed normalization model. Delayed normalization has also been applied to describe dynamics measured using fMRI and ECoG responses in human subjects’ visual cortices^16^. Surprisingly, we found that stimulus-evoked VSDI dynamics qualitatively differ from those measured using these other two methods. Stimulus-evoked VSDI dynamics are near-additive in time beyond brief stimulus presentations, whereas dynamics measured using fMRI and ECoG (broadband signals that exhibit properties consistent with fMRI measures) are substantially sub-additive in time in V1 across all tested stimulus durations.

**Figure 3:**
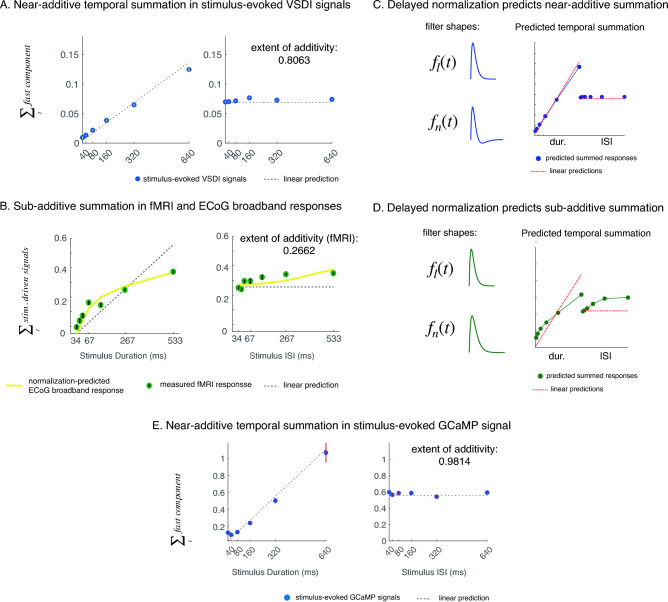
We adopted an additivity test (as in^15^) that was flexible enough to compare temporal additivity across measurement modalities. (**A**) In VSDI, for example, we summed up the stimulus-evoked signals within each condition. Each blue dot represents the sum of the mean response time course for a temporal condition, and the dotted line represents the linear prediction. The blue dots do not significantly deviate from the linear predictions, and the stimulus-evoked VSDI signals are near-additive. The error bars (white, smaller than the dot) indicate the standard error across bootstraps. We further summarized the extent of sub-additivity using a metric developed in^15^ (see “Methods”). The metric is near 1 when the underlying responses are near-additive, and is less than 1 when the responses are sub-additive. (**B**). fMRI data (green) are substantially sub-additive: for example, doubling stimulus duration increased the summed response, but the increase was less than doubled (adopted from Ref.^15^). The predictions of ECoG measurements in V1 to the 12 temporal conditions (based on delayed normalization fit to a set of ECoG data from Ref.^15^ measured using a 500-ms stimulus) are shown in yellow. The prediction of the ECoG data resembled that of the fMRI data. (**C**, **D**) The normalization filters estimated for ECoG data were monophasic (low-pass), whereas the normalization filters for VSDI data tended to be biphasic (band-pass)^16^. Using monophasic versus biphasic normalization filters, we can generate sub-additive versus near-additive temporal dynamics. (**E**) Stimulus-evoked widefield GCaMP responses from behaving macaque V1 (blue dots), like VSDI signals, are also near-additive. The dots represent the summed response extracted from trial-averaged data per stimulus condition, error bars indicate standard error of the response sum extracted from bootstrapped mean data time courses.

To compare additivity across measurement modalities, we adopted a different metric of additivity that can be applied to slower and indirect measurements of neural activity. For example, it is extremely challenging to measure neural filter shapes using fMRI^15,29^, because the BOLD signals are sluggish and are typically sampled every 1–2 s. To compute this new additivity metric, we integrated the time course of the stimulus-evoked VSDI component within each condition to obtain twelve numbers, one for each condition (Fig. 3A, also see “Methods”). If stimulus-evoked dynamics were additive, doubling stimulus duration would double the sum of the response dynamics, and varying inter-stimulus intervals would not change the summed responses^15^. Stimulus-evoked VSDI signals closely follow the additive prediction, consistent with the near-identical linear filters we observed for stimulus duration at or above 80 ms (Fig. 1D). This near-additive response was further confirmed in a second data set, see Fig. S3. We further developed a metric to summarize the extent of temporal subadditivity as in^15^. When the summed responses were near additive, the value of the metric would be close to 1; when the summed responses were supra-additive, the value would be greater than 1; and when the responses were sub-additive, the value would be less than 1 (see “Methods” for more details).

fMRI signals are thought to reflect activities of a local population of neurons, and have been demonstrated to correlate with both local population spike rates and local field potentials (LFP)—slow electrical signals that include, but are not limited to, synaptic potentials^12,14,30,31^. Even though fMRI measurement is coarse in temporal resolution, it can still capture the total response sum (but not dynamics) relating to neural activities evoked by a stimulus presented over different time courses. With fMRI data, a previous study^15^ tested temporal additivity using a similar set of varying duration and ISI conditions. If the underlying neural signals varied additively in time, doubling stimulus duration would double the amplitude of fMRI response. However, cortical responses measured using fMRI were substantially sub-additive. In particular, response amplitude increased but less than doubled when stimulus duration doubled. In the two-pulse temporal conditions, amplitude of the fMRI response to the second stimulus pulse also depends on ISI—response to the second pulse was suppressed for short ISIs and recovered over longer ISIs (Fig. 3B). In addition, the previous study demonstrated that the ECoG response predictions to the same set of stimulus conditions were generally consistent with the fMRI data (Fig. 3B, also see “Methods”). The previous study chose the broadband signals (70–210 Hz range) of the ECoG data for the analysis, because this range of data correlates with local multi-unit activities near the electrodes.

The delayed normalization model paired with different filter shapes can account for both near-additive and sub-additive temporal summation. In VSDI, the measured normalization filter (in the denominator) was biphasic, whereas the normalization filter used to account for ECoG data, which were substantially sub-additive as the fMRI BOLD signals, was monophasic^16,32^. In Fig. 3C and D we demonstrated that with different normalization filter shapes, the model can account for both near-additive and sub-additive temporal summations. Monophasic normalization filter suppressed the input drive over the filter’s entire summation period, whereas for the biphasic filter, the suppressive effect terminated more rapidly.

Temporal additivity in VSDI and in fMRI signals were qualitatively different, and one possible reason could be the distinct sources of the neural signals that these methods sample. VSDI captures the population membrane potential dynamics (mostly sub-threshold), whereas fMRI BOLD signals may mostly reflect spiking activities (supra-thresholds) (e.g.^33^). To examine this possibility, we ran the same temporal experiment using an additional measurement modality—widefield imaging of genetically encoded calcium indicator (GCaMP6f) in V1 of a behaving macaque^11^ (see Fig. S4 for linear and delayed normalization model fit to the GCaMP data). Widefield GCaMP imaging sample neural signals at spatial and temporal scales similar to widefield VSDI, and were shown to approximately relate to the summed local spiking activities in a linear fashion^11^. If the differences between VSDI and fMRI temporal additivities were due to the difference between membrane potential and spiking dynamics, we would expect GCaMP signals to be sub-additive (as the fMRI signals). However, we found that GCaMP signals were more similar to the VSDI measurements, and were even slightly more additive compared to stimulus-evoked VSDI signals (Fig. 3E). See Fig. S5 for GCaMP filter shapes and additional comparison between VSDI and GCaMP signals. Therefore, our GCaMP results suggested that temporal nonlinearities of sub- and supra-threshold population responses in V1 were similar, and that other factors must account for the qualitative difference between VSDI and fMRI dynamics.

## Discussion

Here, we described stimulus-evoked VSDI dynamics using a generalized delayed normalization model. Compared to the linear model, delayed normalization accounts for both higher gain, and slower dynamics in the data at brief stimulus presentations. Additionally, we compared stimulus-evoked dynamics across different methods of population measurements. Surprisingly, we found that stimulus-evoked VSDI and GCaMP dynamics in macaque V1 were near-additive, inconsistent with fMRI signals previously measured in human V1, which were substantially more sub-additive^15^.

In general, deviations from linearity can be partitioned into two types. The deviations can either come from a lack of additivity—doubling the stimulus duration does not double the total amount of responses. Or the deviations can be a result of lacking homogeneity—doubling the stimulus amplitude (i.e. stimulus contrast in this case) does not double the total amount of responses. We used delayed normalization to account for deviations from additivity in stimulus-evoked VSDI signals, and VSDI dynamics were observed to deviate from homogeneity in previous works^11,34^. In this Discussion, we show that the delayed normalization model, with its parameters fitted to the VSDI data, can also qualitatively capture the VSDI deviations from homogeneity.

**Figure 4:**
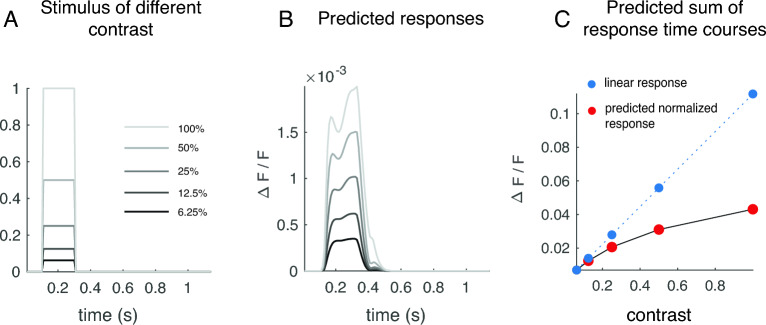
Delayed normalization can qualitatively account for deviation from homogeneity measured in stimulus-evoked  VSDI signals. (**A**) We simulated stimulus-evoked VSDI response to a 200 ms stimulus presentation scaled with different contrasts, ranging from 6.25% to 100%. We used parameters fit to trial-averaged responses to different temporal conditions for this simulation. (**B**) The simulated response time courses for different contrast differ in shape. (**C**) Based on this simulation, doubling stimulus contrast responses results in less than doubled total responses, consistent with previous observations^11,34^.

Sit et al.^34^ observed that when doubling the contrast of a stimulus, the stimulus-evoked VSDI responses increase but are less-than-doubled, and the dynamics of the signal also become faster. To demonstrate that the delayed normalization can encompass these previous observations, we used the model parameters fit to trial-averaged VSDI time courses to generate predictions to a single pulse of stimulus time course (200 ms) presented with different contrasts (Fig. 4A). We generated five different contrast levels, by multiplying the stimulus time course with 5 different scalars, each indicating a contrast ranging from 6.25% to 100%. Predictions of delayed normalization qualitatively agreed with previous observations: the predicted response gains were different at different contrast (an exponential increase in contrast results in a near-linear increase in response amplitude), and shapes of the response dynamics were also different at different contrast levels (Fig. 4B,C). For high-contrast stimuli, the predicted responses exhibited a biphasic pattern, similar to what we observed in the experimental data. At low contrast, the response patterns tended to be monophasic.

When comparing additivity across measurement methods, we found that properties of temporal dynamics measured in stimulus-evoked VSDI and GCaMP signals qualitatively differed from that in fMRI signals. In particular, the stimulus-evoked VSDI and GCaMP signals were near-additive except during a very brief stimulus presentation interval (< 80 ms), whereas fMRI and ECoG broadband signals were sub-additive over a time range of hundreds of milliseconds. This conclusion is independent of how we extract stimulus-evoked dynamics in VSDI and GCaMP measurements (Fig. S6). To illustrate the robustness of our conclusion, we devised additivity tests using other extraction methods from the literature. Existing methods generally assume that the raw VSDI (and GCaMP) time courses consist of two additively combined components—a fast component that reflects stimulus-evoked responses, and a slow “trend” component^18^. Our extraction method, compared to existing methods, assumes a more flexible form for the slow-component, and a delayed normalization model for the fast component (see “Methods”). Stimulus-evoked signals in VSDI and GCaMP extracted using these other methods were slightly noisier, but the conclusion of near-additive temporal summation was robust (Fig. S6). For details and implementation of each extraction model, see “Methods” section.

Delayed normalization was fit to stimulus-evoked VSDI components in our current paper, and the model was fit to ECoG broadband (70 Hz–210 Hz) dynamics in a previous analysis^16^. ECoG broadband signals were thought to better correlate with local population spiking responses. ECoG broadband excluded signals in the low-frequency range (< 70 Hz), whereas VSDI measurements were dominated by the low-frequency signals. This difference in sampling frequency range can potentially provide one explanation to the difference between the dynamics of these two signal types. ECoG broadband, once combined with the lower-range frequencies, could potentially produce signals that are near-additive in time. Second, ECoG broadband signals and VSDI signals have very different dynamics: for a sustained stimulus time course, ECoG broadband signals respond with a transient followed by a decay^16^, whereas VSDI dynamics do not decay for prolonged stimulus presentations. This dynamical difference can potentially contribute to the near-additivity versus sub-additivity in the two signal types. Examining filters fit to both data types, we found that for ECoG broadband signals, both the numerator and the denominator filters in delayed normalization tend to be monophasic. But for VSDI dynamics, the estimated denominator filter tends to be biphasic. In other words, the difference in temporal properties of the two measurements can potentially be accounted by different normalization dynamics. These qualitatively different nonlinearities may reflect species differences or the fact different neural measurements capture distinct aspects of neural computations. Distinguishing between these possibilities awaits to be further examined, but with the delayed normalization model, we were able to quantify this difference, and generate predictions of neural dynamics to any arbitrary stimulus conditions.

## Methods

### Data collection

All procedures have been approved by the University of Texas Institutional Animal Care and Use Committee and conform to NIH standards, and all methods were performed in accordance with ARRIVE guidelines (https://arriveguidelines.org). All the experimental protocols were performed in accordance with relevant guidelines and regulations. The experimental techniques for optical imaging in behaving monkeys were similar to what was described in^10^. In brief, a metal head post was implanted for each animal, and a metal recording chamber was placed over the dorsal portion of V1, a region representing the lower contralateral visual field at eccentricities of 2–5 degrees. We imaged VSDI signals from two monkeys, and GCaMP signals from one monkey. We performed Epi-fluorescence imaging using the following filter sets: GCaMP, excitation 480/20 nm, dichroic 505 nm long-pass, emission filter 520/40 nm; VSDI, excitation 630/20 nm, dichroic 660 nm long-pass, emission filter 690/50 nm). Illumination was obtained with an LED light source (X-Cite120LED) for GCaMP or a QTH lamp (Zeiss) for VSDI imaging. Data acquisition was time locked to the animal’s heartbeat. The sampling frequency for GCaMP imaging was at 20 Hz, and for VSDI imaging it was at 100 Hz.

We collected VSDI data from two animals. The analysis of one animal’s data is presented in the main text, while the analysis of the other, yielding a similar conclusion, is depicted in the Supplement (Fig. S3). We collected GCaMP data from one animal. The detailed analysis of which is shown in the Supplement (Fig. S4).

### Visual stimuli

The stimulus patterns were large-field (8-degree radius) band-passed noise patterns (centered at 3 cycles per degree), and 8 patterns were independently generated, each was presented once for every temporal condition. The patterned stimuli were generated by low-pass filtering white noise at a cutoff frequency of 14 cycles per image, and then thresholded the results. This type of pattern was chosen because (1) it was used in previous studies^15,16^ that we compared our measurement results to; and (2) the patterns activated large V1 responses in humans. The patterns were presented with 12 different temporal conditions. The first six temporal conditions were a single pulse a stimulus presented with various durations (20, 40, 80, 160, 320, 640 ms). The last six temporal conditions were two stimulus pulses with varying inter-stimulus intervals. Each stimulus pulse lasts 160 ms, and the inter-stimulus intervals took values of 20, 40, 80, 160, 320, 640 ms. Notice that the 320 ms single-pulse stimulus is equivalent to a double-pulse stimulus with 0 ISI.

### Data pre-processing

For the VSDI experiments (as well as the GCaMP experiment), we performed three pre-processing steps before extracting stimulus-evoked responses. First, we computed the averaged VSDI response dynamics to two blank conditions (no stimulus). Then we subtracted this averaged time course from VSDI dynamics measured in each stimulus-present trial. This step removed stimulus-independent effects that were shared across all trials (e.g., heart beat artifact, VSDI bleaching, etc.).

Next, for each trial, there was a time delay between stimulus onsets and response onsets. This is because visual signals take time to travel from the eyes to the superficial layer of V1. We can either incorporate this delay into our models, or we can get rid of the delay by shifting response onsets to an earlier time point to match the stimulus onset. Because this delay between response and stimulus onsets is not of interest to our analysis, we took the latter approach, and shifted the response time course forward by 30 ms (for each trial). 30 ms is determined by visualization, as well as on model cross-validation results that we will later describe in the “Method” section.

Third, for each time course, we subtracted the first entry from the entire time course. In other words, we set the beginning of each response time course to 0. As a consequence, the pre-processed VSDI dynamics were relatively similar at the beginning of the trials, and became more diverse towards the end of the trials. GCaMP data pre-processing followed the same steps as VSDI pre-processing.

### ECoG and fMRI data

The ECoG data were re-analyzed from prior work^16^. Two participants were implanted with subdural electrodes for clinical purposes. The study was approved by the Stanford University IRB. For pre-processing, electrodes that had large artifacts identified by the neurologists were excluded from the analysis. The data was down-sampled from the recorded frequency of either 3052 or 1528 Hz to 1000 Hz, and we analyzed the 70–210 Hz broadband ECoG component, because it correlated with local multi-unit activities near the electrodes. The broadband data was obtained via taking a geometric average over 10-Hz bins excluding line frequency within the 70–210 Hz range. At the beginning of each 1-s trial a large field noise image was presented to the observer. Noise images tend to induce a broad-band gamma amplitude increase in the visual cortex, which is thought to correlate with increased spike rate and BOLD signal. The noise pattern was presented for 500 ms, and we used a delayed normalization model to capture the response dynamics. Using the delayed normalization model fit to the ECoG broadband data, we made predictions to the 12 temporal conditions. Then we summed up the prediction in 1200 ms epoch (beginning 200 ms prior to the stimulus onset) for the additivity analysis.

fMRI data were re-analyzed from a prior study^15^ (data were publicly available), and the data was collected over 6 fMRI participants at the Center for Brain Imaging at New York University, and informed consent were obtained from all subjects and/or their legal guardian(s). The experimental stimuli were identical to the ones used in the current study. To extract a single number per stimulus condition for the additivity analysis, the author performed GLM analysis on the raw fMRI data (a regression analysis). A number (regression weight) per trial was then extracted, and was averaged across trials for each stimulus condition.

### Two-component linear model

This model consists of a fast and a slow component, and each component was parameterized by a distinct set of basis functions (Fig. S2B). The fast component was designed to capture stimulus-evoked dynamics, and consists of a filter $f(t)$ convolved with a stimulus time course $s(t)$. The filter was parameterized as a weighted sum of basis functions $\{{f}_{i}(t)\}$, and the number of basis functions was chosen via cross validation. Each basis function was a raised cosine, with the x-axis logarithmically warped^30^, so that the basis functions that were closer to the origin (time 0) vary faster, and the dynamics of the basis functions that were further away from time 0 tend to slow down. This design intended to capture the observations that neural dynamics were fast right after stimulus onsets, and gradually slowed down over time^35^. The fast basis functions cover a duration lasting up to 260 ms (Fig. S2B). We estimated the weights $\{{u}_{i}\}$ for each basis function (described later in the text). The slow component of the model $g(t)$ intended to capture slow VSDI dynamics. The slow component was parameterized using a different set of (slower) basis functions $\left\{{g}_{j}(t)\right\}$, and we estimated weights ${\{v}_{i}\}$ for each of these basis functions. The number of slow basis functions was also chosen via cross-validation, and the slow basis functions spanned the duration of an entire experimental trial (1.2 s; Fig. S2B). In contrast to the fast component, the slow component does not depend on the stimulus, and is not convolved with the stimulus' time course. Overall, the model can be summarized using a single equation:

$$f\left(t\right)*s\left(t\right)+g\left(t\right)= {\sum }_{i=1}^{n}{u}_{i}\left[{f}_{i}\left(t\right)*s\left(t\right)\right]+ {\sum }_{j=1}^{m}{v}_{j}{g}_{j}(t)$$

The set ${\{g}_{j}(t$)} was designed to be different from ${\{f}_{i}\left(t\right)\}$ in three ways to prevent trade-offs between the two components. First, the two sets of basis functions had different time scales, the fast basis functions covered 0.26 s for VSDI signals, and the slow basis functions covered the entire trial duration of 1.2 s. Second, the fast basis functions were convolved with stimulus time courses, and the slow basis functions were directly added to the fast response without interacting with the stimulus. Third, the set ${\{f}_{i}\left(t\right)\}$ had fast-varying basis functions followed by slow ones, whereas the slow-basis functions  ${\{g}_{j}(t$)} started slow and were followed by faster dynamical variations (see Fig. S2B). This is because during the experiment, trial onsets were set to be time-locked to the animal's heartbeat, and we re-set the initial point of each trial of VSDI dynamics to 0. Due to this initial alignment, VSDI time courses tend to have increased variation toward the end of each trial, and this variation can be captured by denser and faster-varying basis functions in ${\{g}_{j}(t$)}.

The GCaMP data was relatively more sluggish compared to the VSDI data. For the GCaMP data, we used a different set of fast basis functions, and the longest duration for the fast basis function is 320 ms. As for the VSDI analysis, we used a set of slow basis functions that covered the entire time course of a 1.35 s trial.

Overall, this approach is simple—the weight for each basis function linearly contributes to the predicted overall VSDI responses and can be estimated via a closed-form least-square minimization. The approach is also flexible—the set of basis functions for the fast component can flexibly approximate common shapes of neural filters, and the set of slow basis functions can flexibly approximate different slow variations in VSDI time courses.

### Two-component delayed normalization model

The two-component delayed normalization model has the same structure as the two-component linear model, and the slow component for this model was implemented the same way as for the two-component linear model.

The fast component consists of a delayed normalization model^16^. The model has a numerator, and a denominator. The numerator consists of a stimulus time course $s\left(t\right)$ convolved with a filter ${f}_{l}\left(t\right)$, which was parameterized using a set of basis functions, as the linear model. The denominator of the model consists of two parts, a non-negative constant $\sigma$ that prevents the denominator from being 0 (otherwise the predicted outcome is undefined), and another filter ${f}_{n}\left(t\right)$ that was convolved with the same stimulus time course $s\left(t\right)$. The filter ${f}_{n}\left(t\right)$ was parameterized as a difference between two Gamma functions (see^16^), this parameterization makes additional assumption on the shape of the ${f}_{n}\left(t\right)$, and better constrains parameter estimations. Overall, the delayed normalization model can be written as

$${r}_{n}(t)=\frac{s\left(t\right)*{f}_{l}(t)}{\sigma +s\left(t\right)*{f}_{n}(t)}$$

The estimated ${f}_{n}\left(t\right)$ was typically slower than the ${f}_{l}\left(t\right)$ in practice, which gave rise to the prediction of slower dynamics at low stimulus contrast or at brief stimulus durations. This is because of the division—the suppressive effect of the denominator was small when stimulus drive (numerator) was also small, and the model dynamics were dominated by the numerator response. If stimulus input became large (e.g. high image contrast), suppressive effect from the denominator would grow, and the denominator started to dominate the dynamics (e.g. response decays sharply after the initial transient).

Using trial-averaged data, we estimated two-component delayed normalization parameters. We separated all of the parameters into two batches—parameters for the delayed normalization, and weights for the additional slow basis functions (for the slow data component). We alternated between the two sets of parameters, and used coordinate descent to find the best fitting model parameters.

### Alternative models to extract stimulus-evoked components in VSDI and GCaMP

#### Linear boxcar with one filter per stimulus

For a stimulus condition, this model assumed the measurement time course could be separated into two additive components, one fast and one slow. The fast component was assumed to be a convolution between a scaled linear filter (parameterized as a gamma function) and a stimulus time course, and the slow component is assumed linear in time, and is modeled as a scalar multiplied by time:

$${c}_{1}s\left(t\right)*{f}_{{c}_{2}}(t)+{c}_{3}t$$

This model is equivalent to a linear model plus a linear trend, which has been commonly used for data pre-processing in the fMRI and the VSDI literature. We fit three model parameters to data averaged across repeats for each stimulus condition, a scalar ${c}_{1}$ for the fast component, a parameter ${c}_{2}$ that governs the shape of the fast filter, and a scalar ${c}_{3}$ for the slow component. Because these parameters nonlinearly interact with each other, we used matlab built-in function *fminsearch.m* to find model parameters.

#### Exponential boxcar with one filter per stimulus

This model shared a similar structure with the previous model, except that the slow dynamics was modeled as an exponential (typically a decay) function, instead of a linear function. This model is expressed as:

$$s\left(t\right)*{f}_{{c}_{1}}\left(t\right)+{c}_{2}{e}^{-{c}_{3}t}+{c}_{4}$$

Notice that in both this and the previous model, we fitted a different filter for each stimulus condition. The other two models in Fig. S6 are the same as these two models, but for each of those models, we assumed that a single fast filter was shared across stimulus conditions.

### Model comparison

We performed leave-one-condition out cross-validation to compare between the two-component linear model and the two-component delayed normalization model. For this cross-validation, we fit the entire model (two-component linear or normalization model) to the trial-averaged left-in conditions (11 out of 12 temporal conditions), and used the fitted model parameters to predict the fast component of the left-out condition. Then we subtracted the predicted fast component from the data time course in the left-out condition, and fitted weights for an additional set of slow basis functions to the remaining slow data variations. To compute variance explained, we compared the sum of the predicted fast component and the fitted slow component to the left-out data time course. Alternatively, we may compare variance explained for the fast component only, by comparing the predicted fast component to the data time course minus the additionally fitted slow component. A similar level of variance-explained can be achieved using this alternative computation.

### Comparing between VSDI, GCaMP and fMRI dynamics

In Fig. 3, we compared the stimulus-evoked component in VSDI and in GCaMP data, to temporal dynamics measured using fMRI^15^ and ECoG^16^. The fMRI experiment shared the same stimulus design and a comparable set of temporal conditions, so we emphasized the comparison between fMRI and VSDI data. The ECoG data were averaged across different stimulus patterns, therefore we only used it to demonstrate that its temporal properties were consistent with that measured in fMRI.

For fMRI data, one number was extracted for each stimulus condition, this is because fMRI hemodynamics were many times slower than neural dynamics, and estimating the time course of neural dynamics would be extremely challenging. The single number extracted for each condition can be viewed as the sum of neural responses for that stimulus condition.

For ECoG, the delayed normalization model was fit to ECoG broadband responses measured in multiple humans’ V1. Broadband responses were thought to relate to LFP, and were correlated with fMRI responses. To make the ECoG predictions comparable to the fMRI measurements, each predicted ECoG time course was summed for a stimulus condition, and an overall scaling factor was fit to the summed responses to make ECoG and fMRI response span the same range.

To make the stimulus-evoked VSDI and GCaMP components comparable to both fMRI and ECoG broadband responses, a two-component normalization model was fit to the trial-averaged measurement time courses. For each condition, we subtracted the estimated slow component away from the data, and summed remaining time courses to get 12 numbers. We compared the 12 numbers obtained from VSDI and GCaMP to the prediction of a linear model, as well as to fMRI and ECoG responses.

We used a metric to summarize the extent of subadditivity in the summed measurement responses, as in^15^. To do so, we fit a scaled power function ${ax}^{c}$ to the summed responses, where $x$ is the total amount of time that a stimulus is presented for each temporal condition, $a$ is a positive scalar, and exponent $c$ summarizes the extent of sub-additivity. When $c$ is close to 1, the summed responses are near-additive, when it is below 1, the summed responses are sub-additive.

### Supplementary Information

Supplementary Figures.

## Data Availability

Published data will be available upon request. Please contact Eyal Seidemann (eyal@austin.utexas.edu) to request data from this study.

## Supplementary Information

The online version contains supplementary material available at 10.1038/s41598-024-63685-6. Code for fitting example data can be found at https://github.com/jingyang-zhou/vsdDynamics_2024.
