# Supplementary material for: Disparate nonlinear neural dynamics measured with different techniques in macaque and human V1
Source: Sci Rep. 2024 Jun 8;14:13193. doi: 10.1038/s41598-024-63685-6 (PMC11162458; doi:10.1038/s41598-024-63685-6)
Supplement: Supplementary file 1 — Supplementary Figures. [file 41598_2024_63685_MOESM1_ESM.pdf]

Supplementary Figures

Figure S1. Slow variations in VSDI signals are stimulus independent.

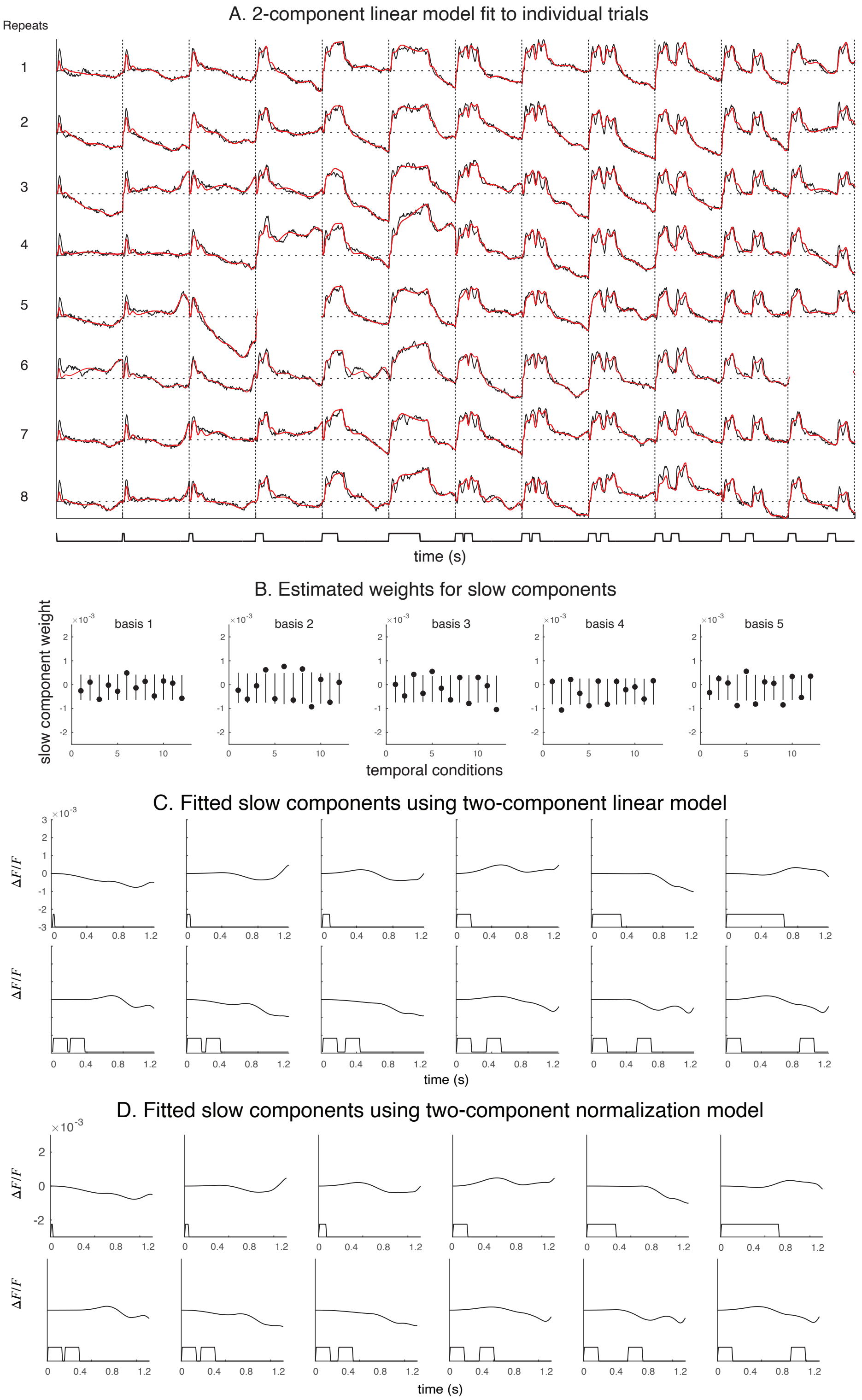

Figure S1. Additional analysis of the VSDI data (from the animal shown in the main text). A. We fitted the two-component linear model (red) to each individual trial (one repeat) of the VSDI data (black). Trials from one repeat of each of the 12 temporal conditions are arranged sequentially and concatenated for display purpose. Rows represent the 8 repeats. Data from two trials were missing due to artifacts detected during the data collection process. For the fitting, a single set of weights for the fast basis functions were shared across all temporal conditions, and a different set of weights for the slow basis functions was fitted to data in each trial. B. We randomized the slow weights estimated for all 12 temporal conditions and all trials, and plotted the 95 percentiles (the vertical bars) of the bootstrapped condition means for all the weights. We compared the slow weights estimated for each condition to these 95 percentiles, and found that most of the estimated slow weights fall within the 95% null range. We conclude that the estimated slow weights are largely independent of the stimulus conditions. C, D. We visualize slow dynamics fit to VSDI time courses (averaged across repeats) using the two-component linear model, and using the two-component delayed normalization model. The estimated slow dynamics using both models do not seem to systematically vary with different stimulus conditions, and seem to be similar between models.

Figure S2. Two-component linear model: schematics.

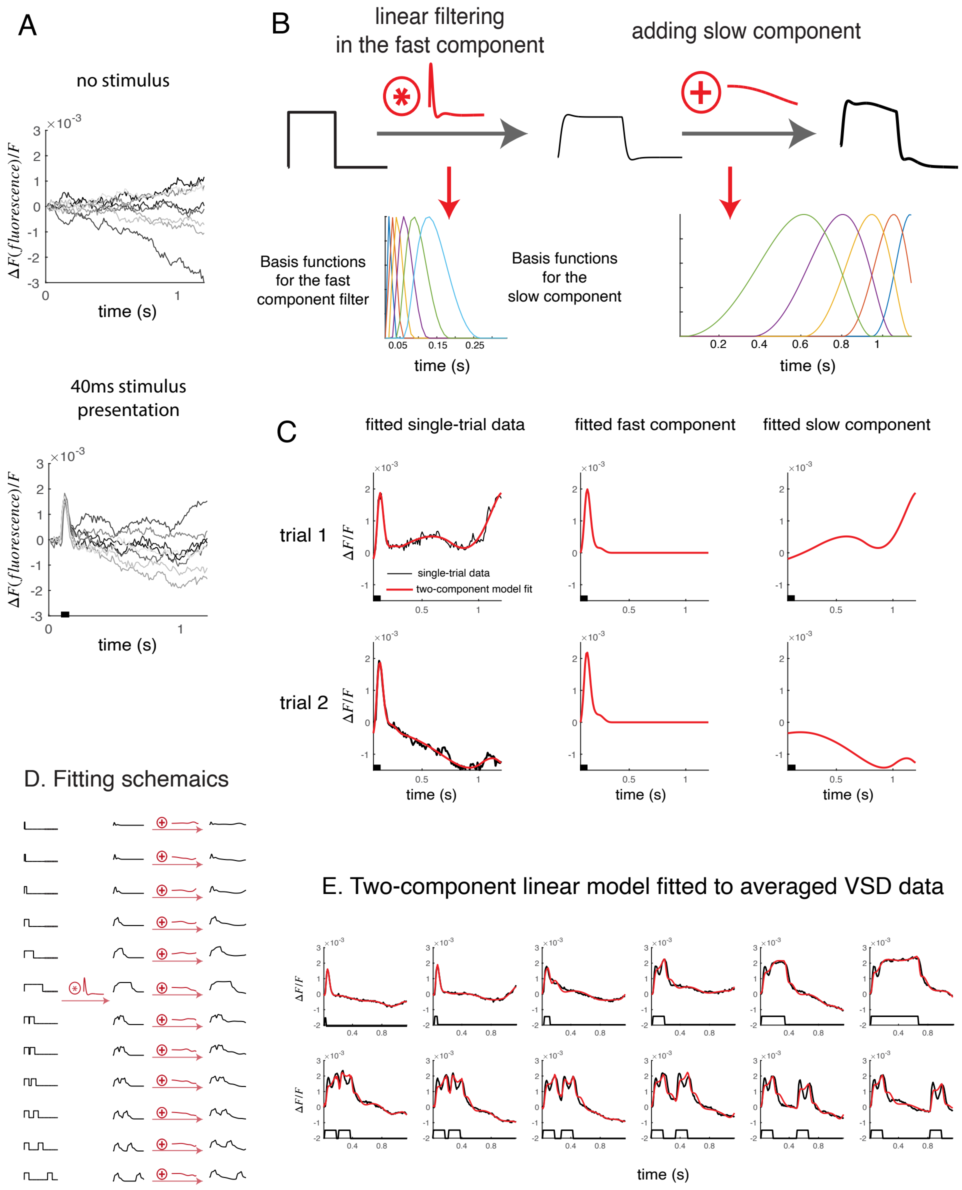

Figure S2. Two-component linear model. A. Different trials of VSDI time courses responding to no stimulus (upper panel), and to a brief stimulus pulse (lower panel). Across different trials, there seems to be a dynamical component that closely tracks the stimulus time course, and is consistent across trials. Additionally, there are other slower dynamics that vary from trial to trial, even at the presentation of the same stimulus time course. B. The two-component linear model consists of a fast component (a filter convolves with stimulus time course), and a slow component that are additively combined with the fast time course. Both the fast filter and the slow component are parameterized using (different) sets of basis functions. C. Fitting the two-component linear model to two example trials. We can see that between the two trials, the fitted fast components are similar, and the fitted slow component vary across trials. D. For

our analysis in Results in the main text, we fitted a single set of fast filters, and distinct set of slow dynamics (one for each temporal condition) to trial-averaged VSDI dynamics. E. The fitted dynamics closely match with the trial-averaged data for each stimulus condition.

Figure S3. An additional set of VSDI data.

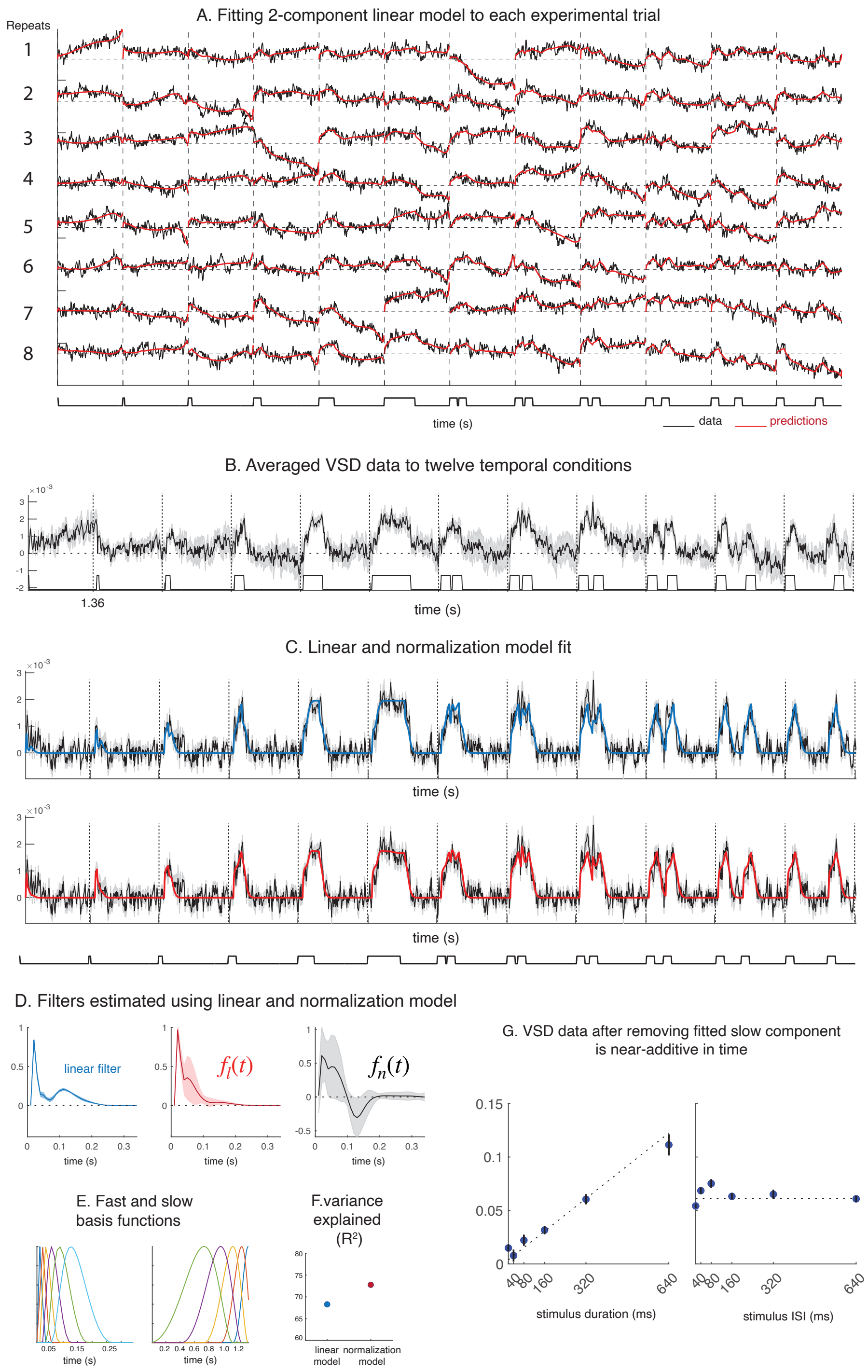

Figure S3. Analyzing an additional set of VSDI data leads to conclusions similar to the main text. A. Two-component linear model fit to each repeat of the additional data set (from a different monkey). B. We analyzed VSDI time courses in response to the twelve temporal conditions, identical to those outlined in

the main text. We plotted the data averaged over eight repetitions of the stimulus presentations (black line). The shaded region represents standard errors generated from bootstrapping the averaged response over the repetitions (sample with replacement, 50 repeats). C. two-component linear and delayed normalization model fit to the additional VSDI data set. We plotted the averaged model fits to the fast components of the VSDI data. C. We visualize filters estimated from this additional dataset. The filters estimated for the linear and the normalization model are qualitatively similar to the filters described in the main text. E. Basis functions used to construct filters and slow components. F. Like in the main text, the normalization model (fitted to the data averaged across bootstraps) out-performed the linear model, summarized using variance explained. G. This additional VSDI data set also exhibits near-additive temporal summation. Error bars represents the standard error (generated from bootstrapping over 50 repeats).

Figure S4. GCaMP data.

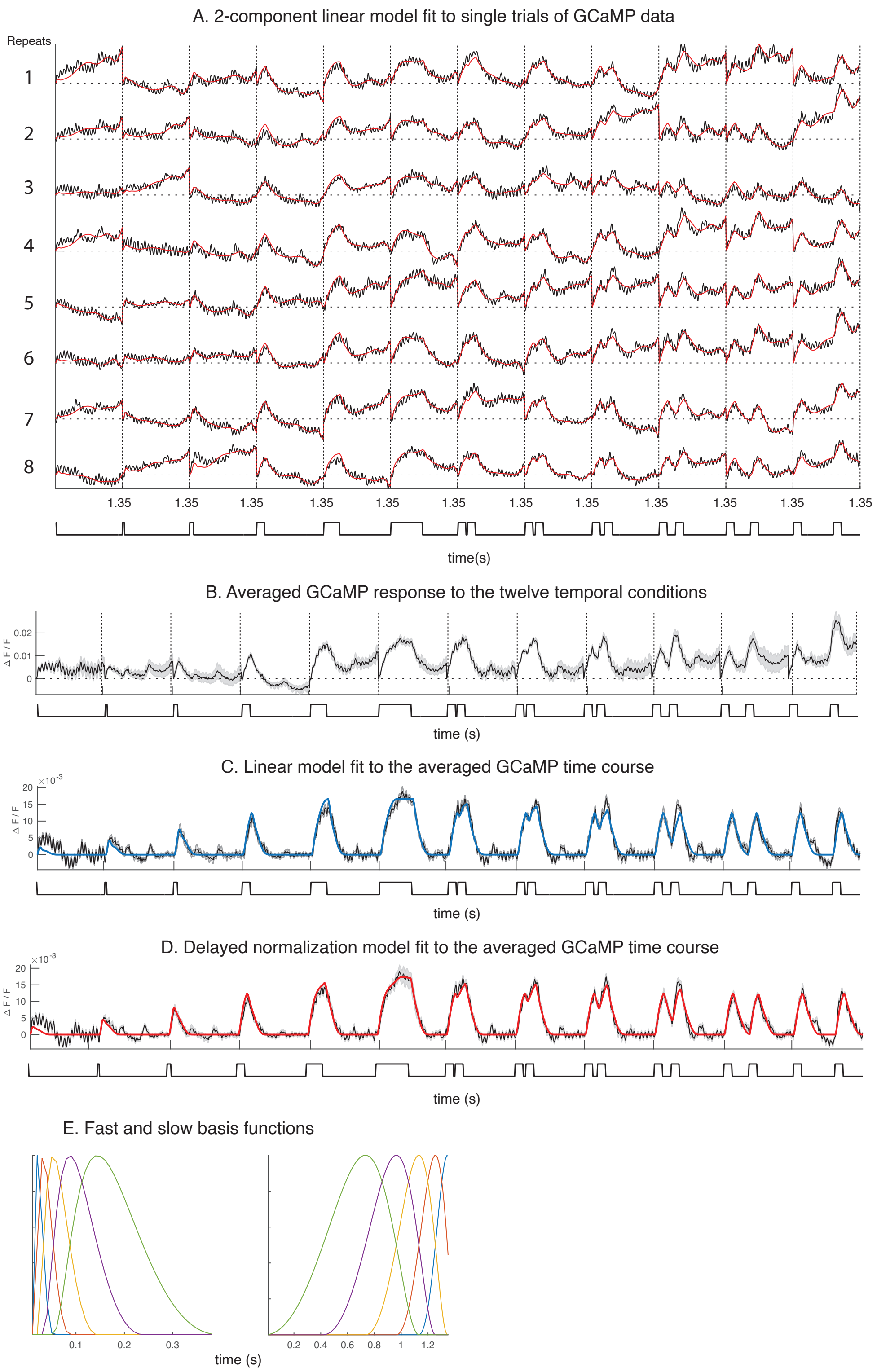

Figure S4. GCaMP response to the same temporal conditions. A. GCaMP responses in individual trials and two-component linear model fit to each repeat of the GCaMP data. B. GCaMP time courses averaged across 50 bootstraps. Shaded area represents the standard error (from bootstrapping the averaged response time courses using sample with replacement and over 50 bootstraps). The stimulus time course is plotted below the response panel. C. Fast component of the linear model (blue) fitted to the fast component extracted from the GCaMP data (same convention as in the main text). D. Fast component of the Delayed normalization model (red) fitted to the fast component extracted from the GCaMP data (same convention as in the main text). E. Basis functions used for the GCaMP analysis.

S5. Comparing VSD and GCaMP filters

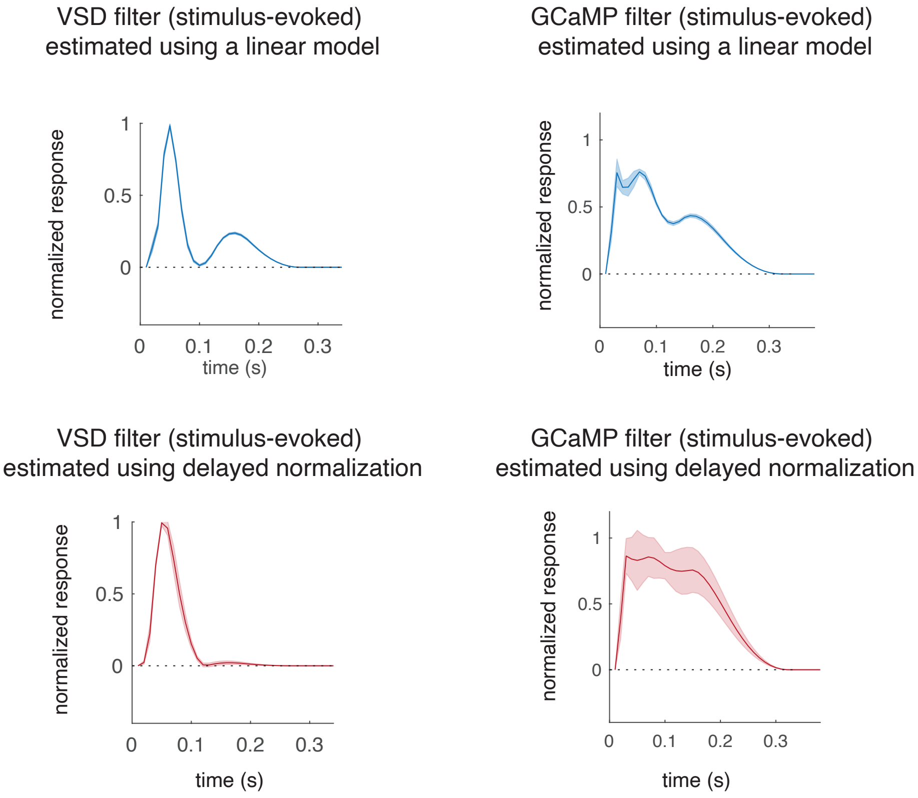

Figure S5. Estimated filters for stimulus-evoked VSD and GCaMP dynamics. For both VSD and GCaMP, filters estimated using two-component delayed normalization seem simpler in shape. This is because part of the filter dynamics (estimated using two-component linear model) is absorbed into the normalization dynamics. The time to peak in each filter seems to be unaffected by which model was used.

S6. Alternative pre-processing methods

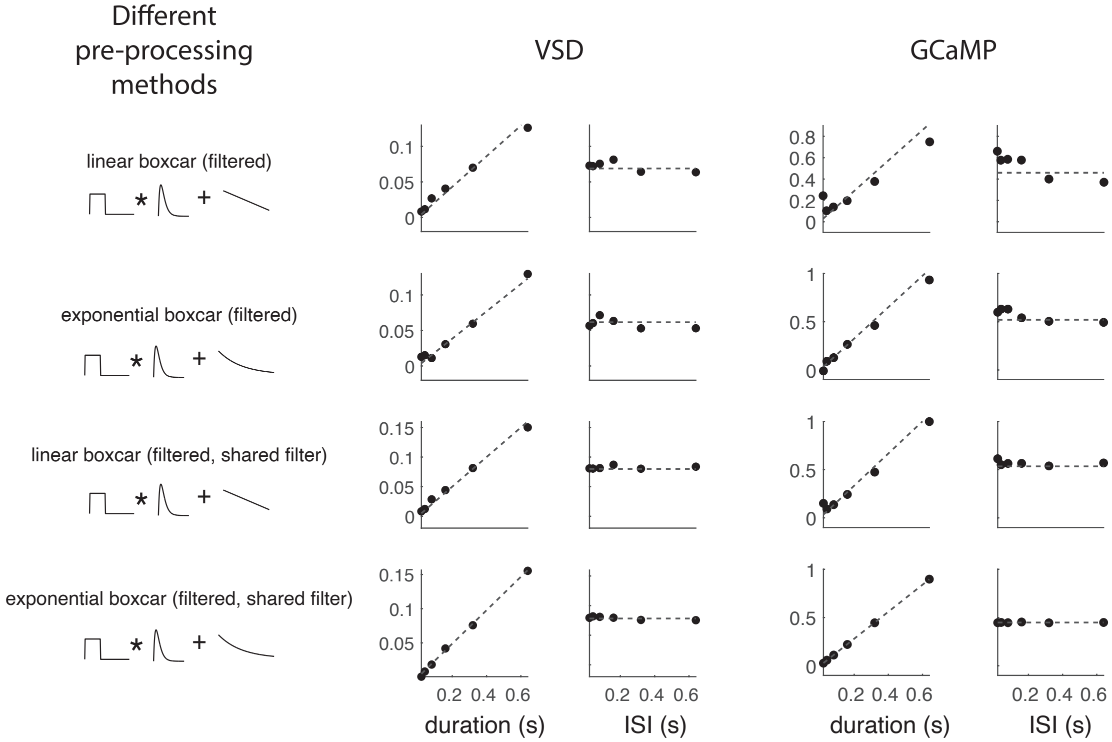

Figure S6. To examine whether near-additive VSD and GCaMP dynamics is a result of our data extraction methods, we used 4 alternative methods to extract stimulus-evoked components from both types of signals, and reached similar conclusions. The first model is a linear boxcar model, and we assume signal dynamics can be approximated by a sum of fast component (a stimulus time course convolved with a filter), and a linear trend. In the second model, we assume the same fast component, but the slow component is approximated with an exponential decay. Notice that in the first two models, a distinct filter was estimated for each stimulus condition. The last two models are the same as previous two models, except that a single filter was estimated for all stimulus conditions.
